# The C-Terminal Sequence of IFITM1 Regulates Its Anti-HIV-1 Activity

**DOI:** 10.1371/journal.pone.0118794

**Published:** 2015-03-04

**Authors:** Rui Jia, Shilei Ding, Qinghua Pan, Shan-Lu Liu, Wentao Qiao, Chen Liang

**Affiliations:** 1 Key Laboratory of Molecular Microbiology and Biotechnology (Ministry of Education) and Key Laboratory of Microbial Functional Genomics (Tianjin), College of Life Sciences, Nankai University, Tianjin, 300071, China; 2 Lady Davis Institute, Jewish General Hospital, Montreal, Quebec, H3T 1E2, Canada; 3 Department of Medicine, McGill University, Montreal, Quebec, H3A 2B4, Canada; 4 Department of Molecular Microbiology & Immunology, School of Medicine, Bond Life Sciences Center, University of Missouri, Columbia, Missouri, 65211–7310, United States of America; German Primate Center, GERMANY

## Abstract

The interferon-inducible transmembrane (IFITM) proteins inhibit a wide range of viruses. We previously reported the inhibition of human immunodeficiency virus type 1 (HIV-1) strain BH10 by human IFITM1, 2 and 3. It is unknown whether other HIV-1 strains are similarly inhibited by IFITMs and whether there exists viral countermeasure to overcome IFITM inhibition. We report here that the HIV-1 NL4-3 strain (HIV-1_NL4-3_) is not restricted by IFITM1 and its viral envelope glycoprotein is partly responsible for this insensitivity. However, HIV-1_NL4-3_ is profoundly inhibited by an IFITM1 mutant, known as Δ(117–125), which is deleted of 9 amino acids at the C-terminus. In contrast to the wild type IFITM1, which does not affect HIV-1 entry, the Δ(117–125) mutant diminishes HIV-1_NL4-3_ entry by 3-fold. This inhibition correlates with the predominant localization of Δ(117–125) to the plasma membrane where HIV-1 entry occurs. In spite of strong conservation of IFITM1 among most species, mouse IFITM1 is 19 amino acids shorter at its C-terminus as compared to human IFITM1 and, like the human IFITM1 mutant Δ(117–125), mouse IFITM1 also inhibits HIV-1 entry. This is the first report illustrating the role of viral envelope protein in overcoming IFITM1 restriction. The results also demonstrate the importance of the C-terminal region of IFITM1 in modulating the antiviral function through controlling protein subcellular localization.

## Introduction

Interferon inhibits virus infection by inducing the expression of hundreds of host genes, known as Interferon-stimulated genes (ISGs), some of which encode antiviral effectors [1]. Among these antiviral proteins are a small protein family called interferon-inducible transmembrane (IFITM) proteins. IFITMs have an average length of 130 amino acids, contain two predicted transmembrane domains and a conserved intracellular domain [2,3]. Although IFITM1 was shown to modestly inhibit vesicular stomatitis virus (VSV) and hepatitis C virus (HCV) more than a decade ago [4,5], the antiviral function of IFITM1, 2 and 3 was established only when their potent restriction of influenza A virus, West Nile virus and Dengue virus was reported in a genome-wide functional screen [6]. Subsequent studies revealed that many viruses are susceptible to IFITM restriction. These include flaviviruses (West Nile virus, dengue virus, and yellow fever virus), filoviruses (Marburg virus and Ebola virus), SARS coronavirus, reovirus, Rift Valley fever virus, human immunodeficiency virus type 1 (HIV-1), jaagsiekte sheep retrovirus (JSRV), and others [6,7,8,9,10,11,12,13] (reviewed in[14,15]). The *in vivo* importance of IFITM proteins in antiviral defense is supported by their protection of mice from influenza A virus infection [16,17,18]. In support of this, single nucleotide polymorphisms in *ifitm3* gene have been shown to associate with pathogenesis severity of influenza A virus infection in humans [16,19].

Humans have five *ifitm* genes, including *ifitm1*, *ifitm2*, *ifitm3*, *ifitm5* and *ifitm10*. They are clustered within a 26.5kb region on chromosome 11 except for *ifitm10* that is located 1.4 Mb downstream [2,20,21]. IFITM5 has a calcium-binding domain at its C-terminal region and is involved in bone mineralization and maturation [22]. IFITM5 has thus also been named bone restricted ifitm-like protein (Bril). IFITM1, IFITM2 and IFITM3 are expressed in a variety of tissues. Their expression is stimulated by type I and type II interferon due to the presence of the interferon stimulation response element (ISRE) in their promoters [2,23]. In addition to their roles in antiviral defense, IFITM1, IFITM2 and IFITM3 have also been reported to participate in antiproliferative signaling, cell adhesion, and oncogenesis (reviewed in [21]). For instance, expression of these three IFITM members is significantly upregulated in colorectal tumor cells concurrent with the activation of the Wnt/beta-catenin signaling pathway [24]. The function of IFITM10 is unknown except that it is the most conserved among all IFITM proteins across many eukaryotic species [20,25].

IFITM1, 2 and 3 proteins differ in their abilities to inhibit different viruses. For example, IFITM3 inhibits influenza A virus more potently than inhibits Marburg and Ebola viruses; in contrast, these latter two viruses are strongly inhibited by IFITM1 [11]. Further, IFITM2 and IFITM3, but not IFITM1, inhibit Rift Valley fever virus [7]. This different restriction profile is likely a result of the sequence divergence between IFITM1, 2 and 3. IFITM2 and 3 share higher sequence homology between each other than with IFITM1 [21]. In this study, we report that human IFITM1 differentially affects the replication of two closely related HIV-1 strains BH10 and NL4–3 and that the viral envelope glycoprotein is responsible for this differential sensitivity to IFITM1 restriction. Interestingly, deleting the C-terminal sequence of IFITM1 allows the mutant to potently inhibit both BH10 and NL4–3 viruses, which highlights the important role of this C-terminal domain in regulating the antiviral function of IFITM1.

## Results

### Human IFITM1 inhibits the replication of HIV-1_BH10_ but not HIV-1_NL4–3_

**Fig 1:**
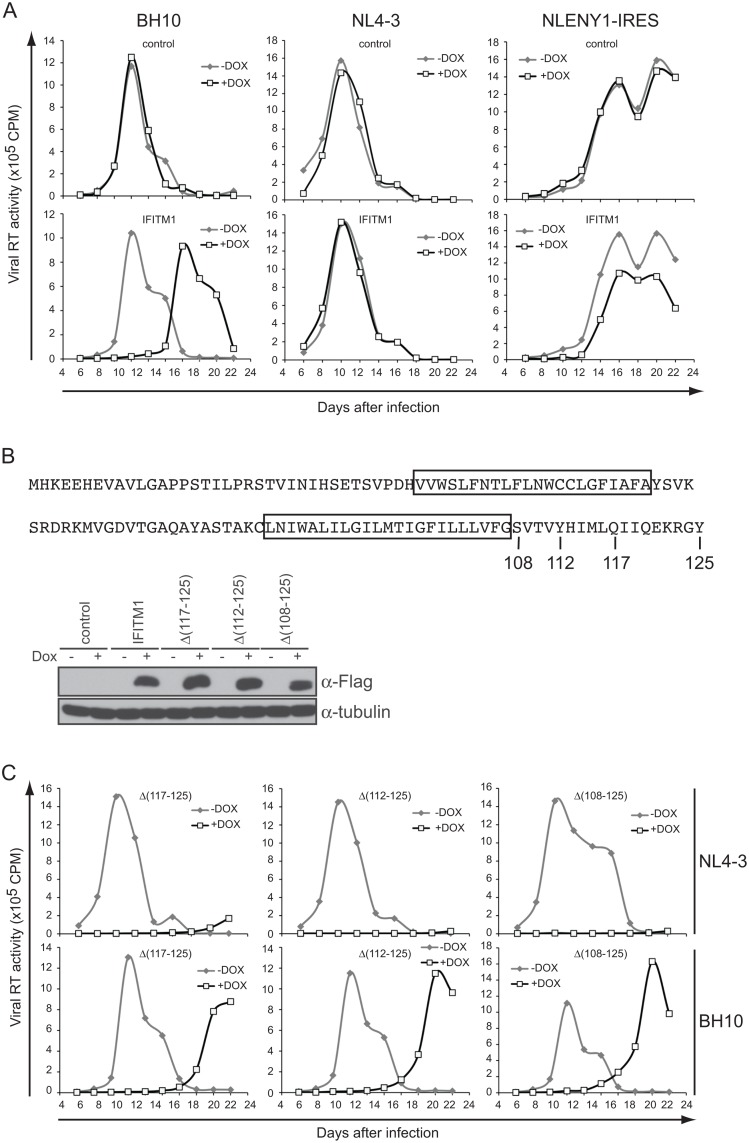
Effect of IFITM1 on HIV-1 replication. (A) SupT1 cells were stably transduced to express IFITM1 under the induction by doxycycline. HIV-1 strains BH10, NL4–3 and NLENY1-IRES (an infectious derivative of NL4–3) were used to infect SupT1 cells with or without doxycycline induction. Virus production was monitored by measuring levels of viral reverse transcriptase (RT) activity in culture supernatants at different time intervals. The infection experiments were performed three times and the result of one representative experiment is shown. Control SupT1 cells were transduced with the empty retroviral vector pRetroX-Tight-Pur. (B) Illustrated is the amino acid sequence of IFITM1. The two predicted transmembrane domains are highlighted. The positions of three deletion mutations are indicated. Both the wild type and mutated IFITM1 have a Flag tag attached to the N-terminus. Levels of the wild type and mutated IFITM1 in stably transduced SupT1 cells were examined by western blotting. An amount of 500 μg/ml doxycycline was used to induce IFITM1 expression. Levels of tubulin were probed as internal controls. (C) Replication of NL4–3 and BH10 in SupT1 cells stably expressing IFITM1 mutants Δ(117–125), Δ(112–125) or Δ(108–125). Virus production was determined by measuring levels of viral RT activity in culture supernatants. A representative result of three independent infections is shown.

We have previously shown that IFITM1, 2 and 3 inhibit the replication HIV-1 strain BH10 in SupT1 cells [10]. When we tested another HIV-1 strain called NL4–3, IFITM1 did not exhibit any inhibitory effect (Fig. 1A), which was in contrast to the marked suppression of HIV-1_NL4–3_ replication by IFITM2 and 3 (S1 Fig.). This observation was further confirmed by the resistance of an HIV-1_NL4–3_-derived virus called NLENY1-IRES to IFITM1 (Fig. 1A).

Human IFITM1 has a relatively long C-terminal region than IFITM2 and 3. We previously showed that IFITM1 mutants lacking this C-terminal sequence still strongly inhibit HIV-1_BH10_ (Fig. 1B and 1C) [10]. Given the resistance of HIV-1_NL4–3_ to the wild type human IFITM1, we expected that HIV-1_NL4–3_ would also be refractory to the C-terminus truncated IFITM1. Surprisingly, IFITM1 mutants Δ(117–125), Δ(112–125) and Δ(108–125) all strongly inhibited HIV-1_NL4–3_ replication (Fig. 1B and 1C). These data suggest that the C-terminal sequence of IFITM1 negatively modulates the anti-HIV-1 function of IFITM1.

### Deleting the C-terminal sequence of IFITM1 leads to inhibition of HIV-1 entry

**Fig 2:**
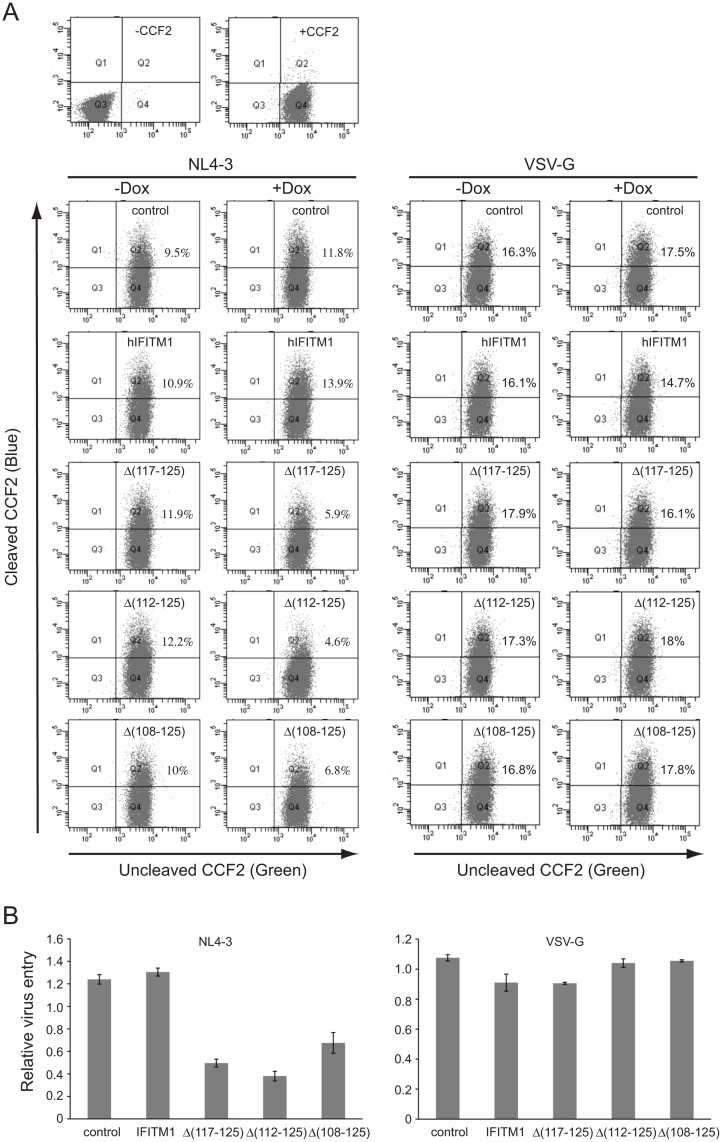
Effects of wild type IFITM1 and its mutants on HIV-1 entry. (A) BlaM-Vpr containing HIV-1_NL4–3_ particles were used to infect SupT1 cells that express wild type IFITM1 or its mutants Δ(117–125), Δ(112–125) or Δ(108–125). Levels of virus entry were determined by monitoring the cleavage of CCF2 by BlaM-Vpr. Similar entry experiments were performed with HIV-1_NL4–3_ that was pseudotyped with VSV G protein. (B) Summary of the results from three independent virus entry experiments.

We have previously shown that, in contrast to IFITM2 and 3, human IFITM1 does not affect the entry of HIV-1_BH10_ [10]. Similarly, no inhibitory effect of IFITM1 on HIV-1_NL4–3_ entry was observed (Fig. 2A). Interestingly, the C-terminal truncated IFITM1 mutants, Δ(117–125), Δ(112–125) and Δ(108–125), diminished HIV-1_NL4–3_ entry by 2 to 3-fold (Fig. 2A and 2B). This inhibition appears to be specific to HIV-1, since neither the WT IFITM1 nor its C-terminal truncations affected entry that was mediated by the G protein of vesicular stomatitis virus (VSV) (Fig. 2A and 2B).

**Fig 3:**
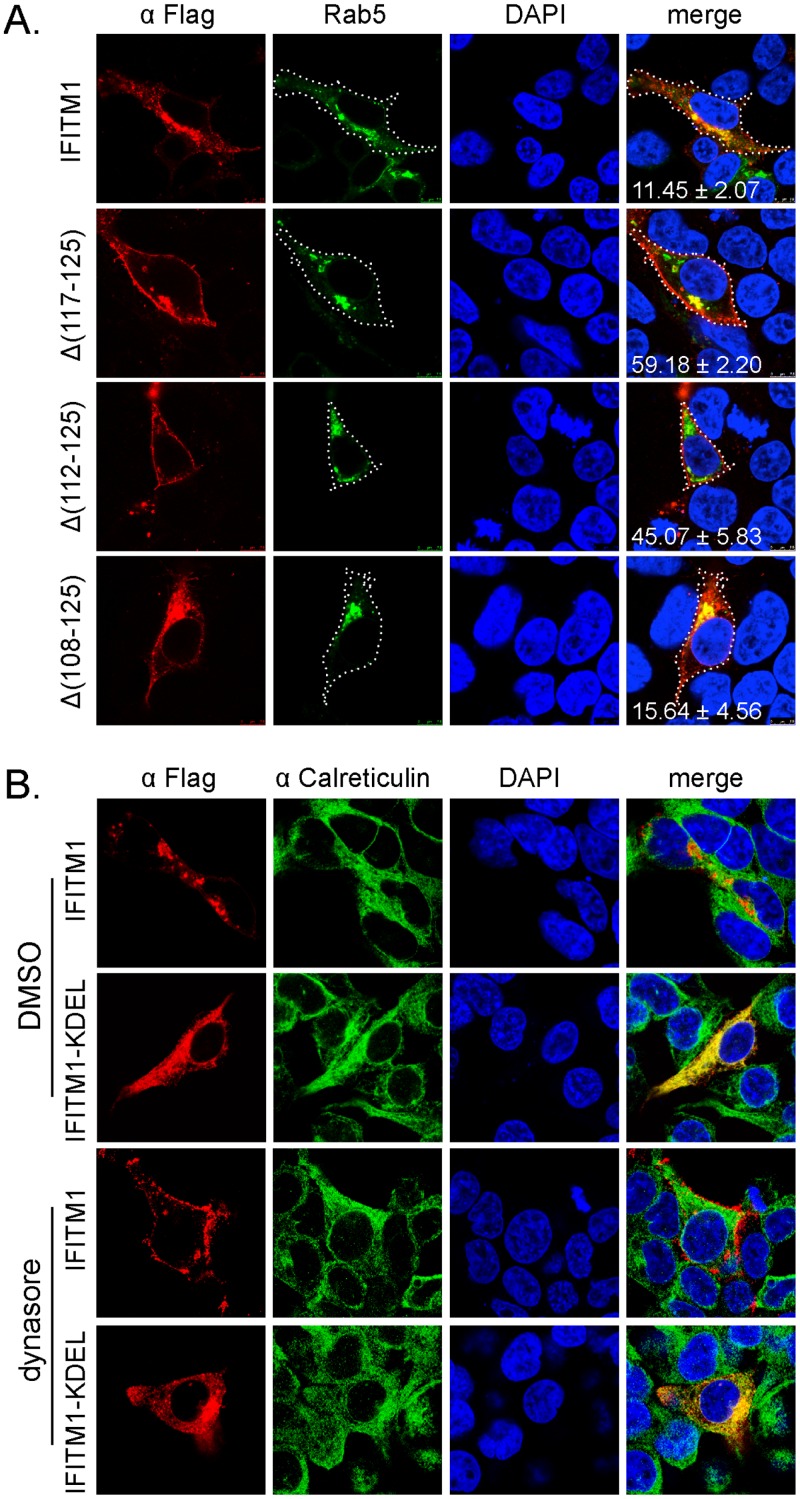
Subcellular localization of wild type IFITM1 and its mutants Δ(117–125), Δ(112–125) or Δ(108–125). (A) HEK293 cells were transfected with plasmid DNA expressing wild type IFITM1 and its mutants Δ(117–125), Δ(112–125) or Δ(108–125) together with Rab5-GFP. Localization of IFITM1 and its mutants were determined by immunostaining with anti-Flag antibody. Nuclei were stained with DAPI. Images shown represent the major subcellular distribution of each protein. The periphery of the immunostained cells was outlined with white dot lines on the basis of the cell morphology observed in phase contrast channel. The fluorescence intensity for IFITM1 and its mutants (in red) at the cell periphery and cell interior was determined using the ImageJ software. The percentage of IFITM1 at the cell periphery was then calculated, and the results obtained from at least 10 cells were shown as mean±standard derivation. (B) HEK293 cells were transfected with plasmid DNA expressing the wild type IFITM1 or its variant IFITM1-KDEL that has the ER retention signal attached to the C-terminus. Cells were either treated with dynasore (160 μM) or DMSO as control. IFITM1 and IFITM1-KDEL were detected by immunostaining with anti-Flag antibody. The endogenous calreticulin was stained with anti-calreticulin antibody. Nuclei were stained with DAPI. Images shown represent the major subcellular distribution of each protein.

In order to understand how the C-terminal sequence of IFITM1 might regulate its ability to impair HIV-1 entry, we examined the cellular localizations of IFITM1 and its C-terminal deletion mutants by immunofluorescence and confocal microscopy. The wild type IFITM1 exhibited a predominant intracellular distribution and showed co-localization with the early endosome marker Rab5 (Fig. 3A).

Removing the last 9 or 14 amino acids from C-terminal sequence re-localized the majority of IFITM1 to the cell periphery (Fig. 3A), indicating that these C-terminus truncated IFITM1 mutants are mostly positioned at the plasma membrane where HIV-1 entry occurs. It was also noteworthy that the Δ(18–125) deletion mutant exhibited an intracellular localization similar to the wild type IFITM1 (Fig. 3A), yet Δ(18–125) markedly diminished HIV-1 entry (Fig. 2). This suggests that the Δ(18–125) mutant may employ a distinct mechanism to deter HIV-1 entry.

We next asked how the C-terminal sequence of IFITM1 modulates protein subcellular distribution. No apparent endocytic sorting signals are present within the sequence 117-QIIQEKRGY-125 of IFITM1, which was deleted in Δ(117–125). One possibility is that this sequence may serve as a retention signal to sequester IFITM1 in the endoplasmic reticulum (ER) or Golgi [26]. This hypothesis is refuted by the results showing that blocking endocytosis with the dynamin inhibitor dynasore caused relocation of IFITM1 to the plasma membrane, as opposed to a IFITM1-KDEL variant that had the ER retention signal KDEL inserted at the C-terminus and exhibited ER localization even in the presence of dynasore (Fig. 3B). These data suggest that IFITM1 is an endocytic protein and that removal of its C-terminal sequence causes its relocation to the plasma membrane where it interferes with HIV-1 entry.

### Mouse IFITM1 diminishes HIV-1 entry

**Fig 4:**
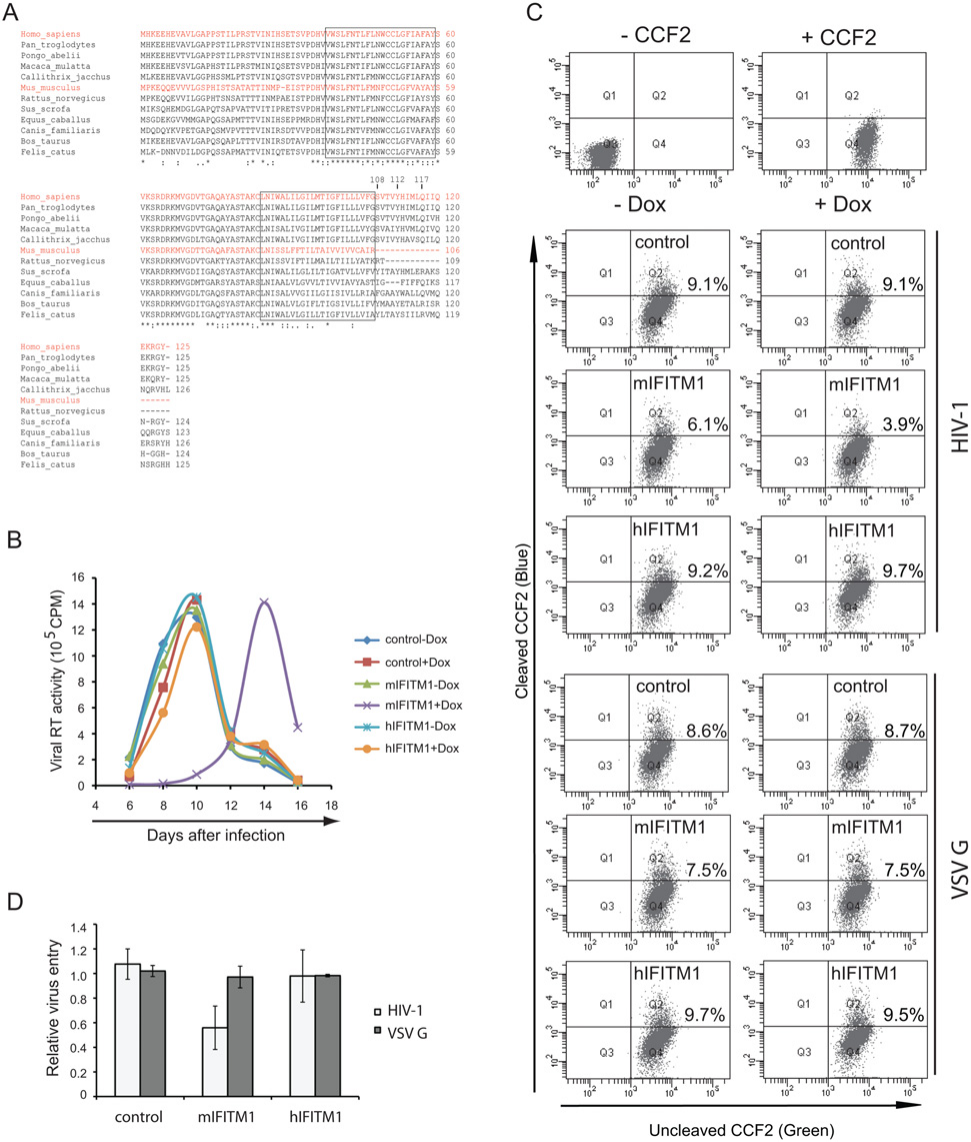
Effect of mouse IFITM1 on HIV-1 infection. (A) Alignment of IFITM1 sequences from different species. Human IFITM1 and mouse IFITM1 are highlighted in red letters. The two predicted transmembrane domains are indicated. (B) Replication of HIV-1_NL4–3_ in SupT1 cells that express mouse IFITM1 (mIFITM1) or human IFITM1 (hIFITM1) under induction by doxycycline. Control cells are transduced with empty retroviral vector pRetroX-Tight-Pur. Growth of viruses was monitored by measuring levels of viral RT activity in culture supernatants at various time intervals. One representative data is shown. (C, D) Effect of mouse IFITM1 on virus entry that was mediated by HIV-1_NL4–3_ Env or VSV G protein. Virus entry is determined by monitoring the cleavage of CCF2. Results of three independent experiments are summarized in the bar graph.

IFITM1 orthologs from different species are highly conserved, except for mouse and rat IFITM1 that have shortened C-termini (Fig. 4A). These two IFITM1 proteins are 19 and 16 amino acids shorter at their C-termini compared to the human IFITM1. This prompted us to test whether they would act like C-terminal truncations of human IFITM1 and inhibit HIV-1 entry. The results of Fig. 4B show that mouse IFITM1 suppressed HIV-1 replication in SupT1 cells. Further, mouse IFITM1 markedly decreased the entry of HIV-1 but not the entry that was mediated by VSV G protein (Fig. 4C and 4D). These data further highlight the importance of the C-terminal sequence of IFITM1 in modulating its antiviral function and also suggest the greater antiviral potential of mouse IFITM1.

### HIV-1 envelope protein modulates virus susceptibility to IFITM1 restriction

**Fig 5:**
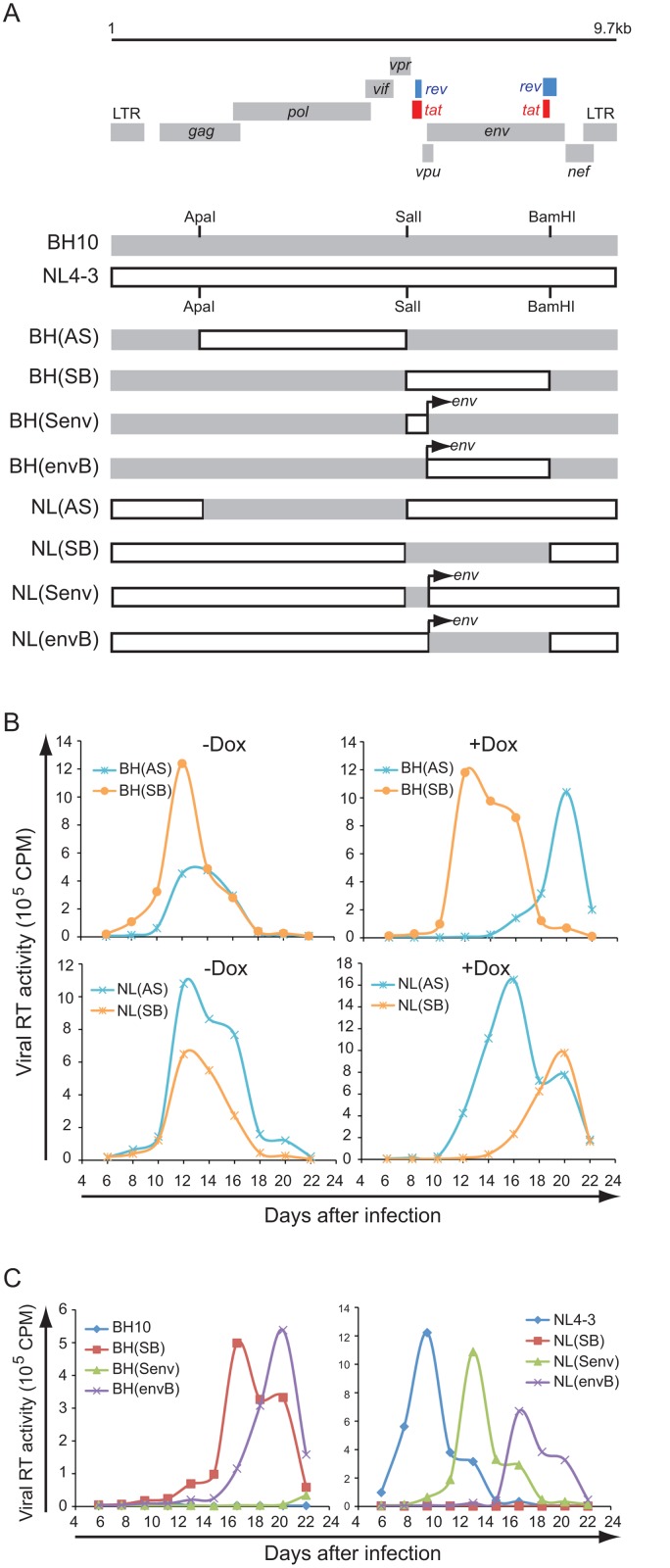
Identification of HIV-1 protein conferring resistance to IFITM1. (A) Illustration of chimeric viruses that were generated by exchanging DNA fragments between HIV-1_BH10_ and HIV-1_NL4–3_. The recognition sites by ApaI, SalI and BamH1 in HIV-1 DNA are shown. AS, between ApaI and SalI sites; SB, between SalI and BamHI sites; Senv, between SalI site and the beginning of Env; envB, between the Env initiation site and BamHI site. (B) Replication of chimeric viruses BH(AS), BH(SB), NL(AS) and NL(SB) in SupT1 cells expressing IFITM1 under induction by doxycycline. (C) Replication of BH10, NL4–3 and their chimeric viruses in IFITM1-expressing SupT1 cells.

We next performed mutagenesis to identify which viral protein(s) accounts for the resistance of HIV-1_NL4–3_ to human IFITM1. To this end, we generated chimeric viruses by exchanging DNA fragments between HIV-1_BH10_ and HIV-1_NL4–3_ and tested which viral DNA fragment(s) bears the genetic information that determines the resistance of HIV-1_NL4–3_ to IFITM1 (Fig. 5A). The results showed that exchange of the 2.7kb DNA fragment, located between the SalI and BamH1 restriction sites (called SB fragment), allowed BH(SB) resistant to IFITM1 and, reciprocally, NL(SB) sensitive to IFITM1 inhibition (Fig. 5B). This SB DNA fragment encodes four viral proteins, Tat, Rev, Vpu and Env. To further determine which viral protein plays the countering role, we exchanged between HIV-1_BH10_ and HIV-1_NL4–3_ only the Env sequence. The results of infection experiments showed that HIV-1_NL4–3_ Env enabled HIV-1_BH10_ to replicate in IFITM1-expressing cells, whereas inserting the Env of HIV-1_BH10_ into HIV-1_NL4–3_ dramatically reduced virus replication in the presence of IFITM1 (Fig. 5C), supporting the role of NL4–3 Env in overcoming IFITM1 inhibition. No significant effect in this regard was observed for the small DNA fragment that is located between the SalI site and the beginning of Env (Fig. 5C).

### HIV-1_NL4–3_ is more efficient than HIV-1_BH10_ in cell-to-cell transmission

**Fig 6:**
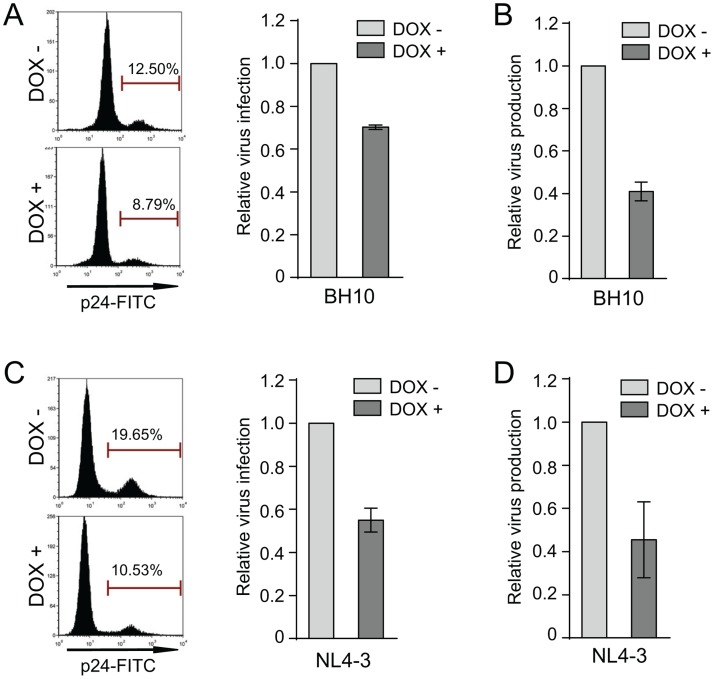
Effect of IFITM1 on HIV-1_BH10_ and HIV-1_NL4–3_ in the one-round infection assay. SupT1 cells were treated with doxycycline (500 μg/ml) for 16 hours to induce IFITM1 expression before infection with HIV-1_BH10_ (A, B) or HIV-1_NL4–3_ (C, D). Forty hours after infection, cells were harvested and stained with FITC-conjugated anti-p24 antibody. Infected cells were scored by flow cytometry (shown in A and C). Amounts of viruses made in the supernatants were determined by ELISA to measure HIV-1 p24/CA (shown in C and D). Results of three independent experiments are summarized in the bar graph.

**Fig 7:**
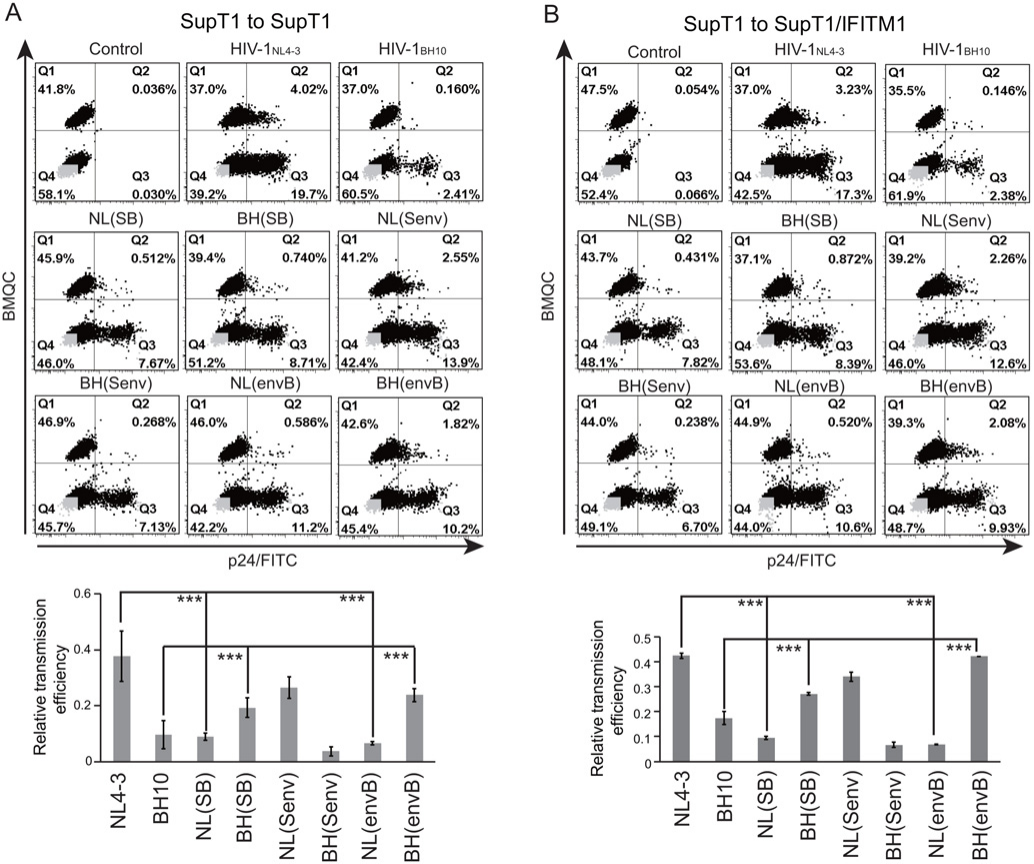
Cell-to-cell transmission of BH10, NL4–3 and their chimeric viruses. (A) Cell-to-cell transmission between SupT1 cells that did not express IFITM1. The donor SupT1 cells were infected with HIV-1, then mixed with target cells that were labeled with cell tracker BMQC. Infected cells were monitored by immunostaining viral p24 using FITC-conjugated anti-p24 antibody. The infected target cells are scored in the Q2 window of the flow cytometry plots. Relative transmission efficiency was calculated by dividing the percentage of infected target cells (Q2/(Q1+Q2)) with that of infected donor cells (Q3/(Q3+Q4)). Results of three independent transmission experiments are summarized in the bar graph. (B) Cell-to-cell transmission from control SupT1 cells that do not express IFITM1 to SupT1 cells that express exogenous human IFITM1. The control SupT1 cells were first infected with HIV-1 and used as the donor cells to transmit viruses to target cells. Transmission efficiency was calculated as described above. *** denotes p value <0.01.

One mechanism behind the IFITM1 inhibition of HIV-1_BH10_ is the reduction in Gag/p24 levels in the infected cells as well as the diminution in virus production (Fig. 6A and 6B) [10]. Similar defects were also observed for HIV-1_NL4–3_ infection of IFITM1-expressing SupT1 cells (Fig. 6C and 6D), which indicates that HIV-1_NL4–3_ escapes IFITM1 in the spread infection without the need to restore this decrease in Gag/p24 expression. We recently reported that HIV-1_BH10_ became resistant to IFITM1 restriction in the spread infection through acquiring mutations in viral Env and Vpu proteins that together enhance the virus transmission between cells [27]. In order to test whether the same mechanism supports the resistance of HIV-1_NL4–3_ to IFITM1, we compared the cell-to-cell transmission efficiency of HIV-1_BH10_ and HIV-1_NL4–3_. Indeed, HIV-1_NL4–3_ was 3-fold more efficient than HIV-1_BH10_ in transmitting between SupT1 cells (Fig. 7A). This phenotype was readily reversed when the Env sequence was exchanged between HIV-1_BH10_ and HIV-1_NL4–3_, either by exchanging the SB fragment or the Env sequence alone (Fig. 7A). Similar observations were made for virus transmission from control SupT1 cells to SupT1 cells expressing IFITM1 (Fig. 7B). Collectively, these data suggest that the Env protein of HIV-1_NL4–3_ mediates greater cell-to-cell transmission, a property that may allow HIV-1 to escape IFITM1 inhibition under the virus spread infection condition.

## Discussion

This study was based on the observation that two closely related HIV-1 subtype B strains BH10 and NL4–3 were differentially inhibited by human IFITM1 in virus spread infection. This finding allowed us to identify the viral component(s) in NL4–3 that gave rise to IFITM1 resistance. Results of mutagenesis and virus replication studies revealed an essential role of viral Env protein in HIV-1_NL4–3_ evasion from IFITM1. Exchanging the Env sequences between BH10 and NL4–3 reversed the susceptibility of the parental strains to IFITM1 inhibition. This role of HIV-1 Env protein in determining viral sensitivity to IFITM1 corroborates our recent report showing that HIV-1_BH10_ was able to develop resistance to IFITM1 through mutating Env and Vpu [27]. There are precedents illuminating the function of viral Env in overcoming host restriction. One example is the HIV-2 Env that antagonizes tetherin [28]. In addition, HIV-1 Env has been shown to regulate the establishment of latent infection in resting CD4+ T cells [29], likely through modulating the functionality of certain cellular pathways [30]. In the case of IFITM1, it remains to determine whether HIV-1 Env serves as the viral target of IFITM1 or Env acts as a viral antagonist to counter IFITM1.

Although IFITM1 does not affect the entry of either HIV-1_BH10_ or HIV-1_NL4–3_, production of both viruses is diminished in the one-round infection assay, which alludes to inhibition of a post-entry step of HIV-1 infection by IFITM1. To our surprise, in spite of being inhibited to similar degrees in the one-round infection assay, only HIV-1_BH10_, but not HIV-1_NL4–3_, is crippled by IFITM1 in the long-term virus replication assay (Fig. 1). These seemingly conflicting observations could be reconciled by the major difference between the one-round infection and the long-term infection, i.e. the latter infection mode involves virus cell-to-cell transmission that actually dominates HIV-1 transmission over infection by free virus particles [31]. It is thus possible that the superiority of HIV-1_NL4–3_ over HIV-1_BH10_ in replicating in IFITM1-expression SupT1 cells, despite that both viruses are equally impaired by IFITM1 in the one-round infection, might be a result of the greater ability of HIV-1_NL4–3_ to transmit between cells. Indeed, we found that HIV-1_NL4–3_ was 3-fold more efficient in transmission from cell to cell than HIV-1_BH10_. HIV-1_NL4–3_ has therefore utilized its strong cell-to-cell transmission to compensate for the deficiency in virus production caused by IFITM1.

The efficiency of cell-to-cell transmission is determined by viral Env protein. Inserting the Env sequence of HIV-1_BH10_ into HIV-1_NL4–3_ diminishes cell-to-cell transmission of the parental virus. Reciprocally, HIV-1_NL4–3_ Env protein increases cell-to-cell transmission of HIV-1_BH10_ by 3-fold. Concurrent with the change in cell-to-cell transmission, this exchange in Env sequences also switches the sensitivity of HIV-1_BH10_ and HIV-1_NL4–3_ to human IFITM1. One possible scenario is that HIV-1 Env protein may modulate virus sensitivity to human IFITM1 restriction by virtue of its ability to mediate and regulate virus cell-to-cell transmission. The Env sequences of HIV-1_BH10_ and HIV-1_NL4–3_ differ at 23 amino acid positions. Changing each of these 23 amino acids in HIV-1_BH10_ Env to the counterpart in HIV-1_NL4–3_ did not increase the resistance of HIV-1_BH10_ to human IFITM1 (data not shown). It can be conceived that two or more of these 23 amino acids in Env are required to generate resistance to IFITM1.

Human IFITM1 does not affect the entry of either HIV-1_BH10_ or HIV-1_NL4–3_ (Fig. 2) [10]. Interestingly, IFITM1 gains the ability to inhibit HIV-1 entry when its C-terminal sequence is truncated (Fig. 2). In agreement with this observation, C-terminal truncation enhances the ability of IFITM1 to promote the infection of Coronavirus OC43 [32]. This gained function appears to be virus specific, since the truncated IFITM1 does not affect virus entry mediated by VSV G protein (Fig. 2). One possible explanation of this differential inhibition is that HIV-1 and VSV adopt different pathways for entry. HIV-1 entry is pH-independent and occurs at the plasma membrane, whereas VSV enters cells in the endosomes of low pH [33,34]. In support of this scenario, removal of the last 9 amino acids from the C-terminus of human IFITM1 relocates this protein from intracellular sites to cell periphery (Fig. 3). This cellular location-dependent inhibition phenomenon was previously reported for IFITM3. Deleting the endocytic sorting signal 20-YEML-23 in IFITM3 led to redistribution of IFITM3 to the plasma membrane and the consequent loss of inhibition of influenza A virus entry that takes place at late endosomes [35,36,37,38]. In addition to the regulation by their own sequences, subcellular distribution of IFITMs can also be modulated by other factors. For example, compared to its intracellular localization in 293T cells as shown in this study, the exogenous IFITM1 has been observed at the plasma membrane in A549 cells [39]. In addition to the cell type-dependent effect, other factors including expression levels and treatment with interferon may also affect where IFITM proteins are localized in cells.

It is unclear how the C-terminal sequence modulates the subcellular distribution of IFITM1, especially in light of the recent report showing that IFITM1 C-terminal domain is extracellular [39]. One mechanism by which extracellular/luminal protein domain regulates protein subcellular localization is the ER-luminal retention signal [26]. Our data rule out the presence of ER-luminal retention signal within the C-terminal region of IFITM1, because inhibiting endocytosis with the dynamin inhibitor dynasore redistributes IFITM1 to the plasma membrane, but the same treatment fails to do so to an IFITM1 variant with the exogenous ER retention signal inserted at the C-terminus (Fig. 3B). Alternatively, deleting the C-terminal sequence may change the membrane topology of IFITM1 and, as a result, disturb its subcellular localization.

It remains to be determined if other HIV-1 strains are also differentially inhibited by human IFITM1. We suspect that the chance of finding human IFITM1-sensitive HIV-1 strains, either lab-adapted or naturally-existing or circulating, would be low if IFITM1 exerts selection pressure *in vivo* on HIV-1 replication. Since both HIV-1_BH10_ and HIV-1_NL4–3_ are strains that have been propagated in cultured human T cell lines, they have adapted to the *in vitro* culture conditions and as a result, they could have lost the original viral sequences that confer resistance to IFITM1, which may have happened to HIV-1_BH10_. We envision that since HIV-1 has never encountered mouse IFITM1, most, if not all, of the HIV-1 strains would be sensitive to the mouse IFITM1 inhibition. Work is ongoing to examine the effect of IFITM proteins from different species on other strains of HIV and different lentiviruses.

## Materials and Methods

### Plasmid DNA and antibodies

The HIV-1 proviral DNA clone HIV-1_NL4–3_ was obtained from the NIH AIDS Research and Reference Reagent Program. The NLENY1-IRES DNA is a derivative of HIV-1_NL4–3_ [40]. The NLENY1-IRES-ES DNA is similar to NLENY1-IRES except for insertion of stop codon at the beginning of *env* gene and is therefore Env-negative [40]. Rab5-GFP was a gift from Stephen Ferguson and Michel Tremblay. The Tet-IFITM1 DNA construct has the cDNA of human *ifitm1* gene inserted into the pRetroX-Tight-Pur vector. The IFITM1 mutants Δ(117–125), Δ(112–125) and Δ(108–125), which have different lengths of the C-terminal sequence (illustrated in Fig. 1), have been previously reported [10]. The ER retention signal KDEL was added to the C-terminus of IFITM1 by a PCR-based method. The cDNA clone of mouse *ifitm1* was purchased from ATCC, amplified by PCR, and cloned into pRetroX-Tight-Pur to generate Tet-mIFTM1. Like all human IFITM1 constructs, a Flag tag was added to the N-terminus of mIFITM1 to facilitate detection by western blotting. All these DNA clones were verified by sequencing. The anti-Flag antibody was purchased from Sigma, anti-tubulin antibody from Santa Cruz Biotechnology, FITC-conjugated anti-HIV-1 p24 antibody from Beckman, anti-calreticulin antibody from Abcam, and BMQC from Invitrogen. The HEK293 cells and SupT1 cells were originally obtained from ATCC.

### Generating stably transduced cell lines

SupT1 is a human CD4+ T cell line. They are cultured in RPMI1640 medium supplemented with 10% fetal bovine serum (FBS), 2 mM L-glutamine, 100 U of penicillin/ml and 100 μg of streptomycin/ml. In order to stably transduce SupT1 cells, we first prepared retrovirus stocks by transfecting the GP-2 packaging cells (Clontech) with the pRetroX-Tight-Pur vector together with the pCMV-VSV-G plasmid (expressing the G protein of vesicular stomatitis virus (VSV)). The viruses were then used to infect SupT1 cells together with another retrovirus carrying the rtTA activator gene (Clontech). The infected SupT1 cells were cultured in the RPMI1640 medium supplemented with 10% tetracycline-free FBS (Clontech), 2 μg of puromycin/ml and 1 mg G418/ml to select for stably transduced cells.

### Virus infection

HIV-1 stocks were produced by transfecting human embryonic kidney 293T (HEK293T) cells with HIV-1 proviral DNA clone using Lipofectamine2000 (Invitrogen). The culture supernatants were clarified by centrifugation at 3,000 rpm in a Beckman bench-top centrifuge at 4°C for 30 min. The viruses were aliquoted, used immediately or stored at -80°C for future use. Amount of viruses was determined by measuring viral p24(CA) in ELISA.

SupT1 cells stably transduced with the Tet-IFITM retroviral vectors were treated with doxycycline (0.5 mg/ml) before exposure to HIV-1 equivalent to 20 ng viral p24(CA) per 2x10^6^ cells. Virus growth was monitored by measuring viral reverse transcriptase activity in the supernatants at different time intervals.

### HIV-1 virion fusion assay

The experiments were performed as described in [41]. Briefly, two virus stocks were first produced by transfecting the HEK293T cells using either HIV-1_NL4–3_ proviral DNA or the NLENY1-IRES-ES DNA (Env-) and the pCMV-VSV-G DNA together with pCMV-BlaM-Vpr. The virus stocks were named HIV-1_NL4–3_/BlaM-Vpr that has HIV-1 envelope protein, and NLENY1/VSV-G/BlaM-Vpr that has the VSV-G protein as the envelope, respectively. The amount of viruses in each stock was determined by measuring viral p24(CA) in ELISA. The SupT1 cell lines were first treated with doxycyline (0.5 mg/ml) for 16 hours, then infected with either HIV-1NL4–3/BlaM-Vpr (an amount equivalent to 100 ng viral p24(CA)) or NLENY1/VSV-G/BlaM-Vpr (an amount equivalent to 50 ng viral p24(CA)) by spinnoculation at 1,800 x g for 45 min at room temperature. Following a 2-hour incubation at 37°C, cells were washed twice with CO_2_-independent medium, then incubated in the loading solution at room temperature for one hour. The loading solution contained 2 μl of CCF2/AM (1 nM) and 8 μl of 0.1% acetic acid (containing 100 mg/ml Pluronic-F127surfactant) in 1 ml of CO_2_-independent medium. After washing with development media (containing 10 μl of probenecid (250 mM) and 100 μl tetracycline-free FBS in 1 ml CO_2_-independent medium, Invitrogen), cells were kept in dark at room temperature for 16 hours before being fixed in 1% paraformaldehyde and analyzed by flow cytometry.

### Virus cell-to-cell transmission assay

First, SupT1 cells were infected with HIV-1 particles bearing VSV-G protein to enhance infection efficiency. Forty hours post infection, the infected cells (designated as donor cells) were washed with complete medium, then mixed with equal number of un-infected SupT1 cells (designated as target cells), which had been labeled with a cell tracker BMQC (Invitrogen). After 8 hours, the cell mixtures were fixed with 1% paraformaldehyde and permeabilized with 0.1% Triton X-100. After staining with FITC-conjugated anti-HIV-1 p24 antibody, the p24-positive donor and target cells were scored by flow cytometry.

### Immunofluorescence microscopy

HEK293 cells were seeded onto poly-lysine-coated coverslips and transfected with the indicated IFITM1 DNA constructs. Cells were fixed in 4% paraformaldehyde in phosphate buffered saline for 10 min at room temperature, permeabilized with 0.1% Triton X-100 for 10 min, and incubated with blocking buffer containing 3% BSA and 6% skim milk. Cells were then stained with indicated primary antibodies (1:100 dilution) for 2 hours at room temperature, followed by staining with FITC or TRITC conjugated secondary antibodies (1:100 dilution). The nuclei were stained by DAPI. Dynasore treatment was performed by incubating cells with 160 μM dynasore for 1 hour at 37°C prior to fixation. Images were acquired with Leica TCS SP5 laser scanning confocal microscope using 100x objectives with sequential-acquisition setting. All the images were obtained using the laser power and digital gain below saturation at a resolution of 1024 x 1024 pixels (8 bit). ImageJ software was used for post-acquisition measurement of the fluorescent intensity.

## Supporting Information

S1 Fig Effect of IFITM2 and 3 on the replication of HIV-1_NL4–3_. SupT1 cells were treated with doxcycyline to induce the expression IFITM2 and 3 before being exposed to HIV-1_NL4–3_. Virus production was determined by measuring viral reverse transcriptase activity in culture supernatants at various time intervals.
(EPS)
